# Supplementary material for: Prevalence of Type 2 Diabetes and Its Association with Added Sugar Intake in Citizens and Refugees Aged 40 or Older in the Gaza Strip, Palestine
Source: Int J Environ Res Public Health. 2020 Nov 19;17(22):8594. doi: 10.3390/ijerph17228594 (PMC7699327; doi:10.3390/ijerph17228594)
Supplement: Supplementary file 1 [file ijerph-17-08594-s001.pdf]

Table S1. Characteristics of study participants according to population; (Refugees and Citizens). [a]

Variable	All *n* = 1000	Refugees *n* = 422	Citizens *n* = 578	*p*-Value
Age, years	59.2 (7.5)	59.4 (7.6)	58.9 (7.5)	0.282
Women	468 (46.8%)	197 (46.7%)	271 (46.8%)	0.949
Study regions				0.698
North	220 (22.0%)	92 (21.8%)	128 (22.2%)	
Gaza	122 (12.2%)	47 (11.1%)	75 (12.9%)	
Midzone	214 (21.4%)	96 (22.8%)	118 (20.4%)	
Khan Younis	223 (22.3%)	89 (21.1%)	134 (23.2%)	
Rafah	221 (22.1%)	98 (23.2%)	123 (21.3%)	
Family size	6.9 (1.5)	6.9 (1.5)	6.9 (1.5)	0.323
Married	844 (84.4%)	359 (85.1%)	485 (83.9%)	0.849
Education				0.658
Low	451 (45.1%)	186 (44.1%)	265 (45.9%)	
Moderate	295 (29.5%)	131 (31.0%)	164 (28.4%)	
High	254 (25.4%)	105 (24.9%)	149 (25.7%)	
Employed	117 (11.7%)	52 (12.3%)	65 (11.3%)	0.601
Household income (NIS)				0.439
<500	51 (5.1%)	25 (5.9%)	26 (4.5%)	
500-<1000	41 (4.1%)	20 (4.7%)	21 (3.6%)	
1000-1500	48 (4.8%)	18 (4.3%)	30 (5.2%)	
>1500	6 (0.6%)	1 (0.3%)	5 (0.9%)	
No constant income	854 (85.4%)	358 (84.8%)	496 (85.8%)	
Having family history of NCDs [b]	468 (46.8%)	192 (45.5%)	276 (47.8%)	0.481
Type 2 Diabetes	452 (45.2%)	193 (45.7%)	259 (44.8%)	0.546
Physical activity				0.717
Low	654 (65.4%)	270 (64.0%)	384 (66.4%)	
Moderate	247 (24.7%)	108 (25.6%)	139 (24.1%)	
High	99 (9.9%)	44 (10.4%)	55 (9.5%)	
T2D Medications (For T2D patients)				0.633
Oral hypoglycaemic drugs	225 (49.7%)	101 (52.3%)	124 (47.8%)	
Insulin	171 (37.8%)	71 (36.7%)	100 (38.6%)	
Both	56 (12.4%)	21 (10.9%)	35 (13.5%)	
Current cigarette's smoking	446 (44.6%)	194 (45.9%)	252 (43.6%)	0.456
BMI, kg/m^2	28.2 (3.7)	28.3 (3.6)	28.1 (3.7)	0.902
Waist circumference, cm	105.0 (14.7)	106.3 (14.8)	104.0 (14.6)	0.797
Fasting glucose, mmol/l	9.4 (4.7)	9.6 (4.7)	9.4 (4.6)	0.638
HbA$_{1c}$, mmol/mol	48.4 (18.8)	48.2 (14.8)	48.6 (18.9)	0.327
HbA$_{1c}$, %	6.6 (1.7)	6.5 (1.6)	6.6 (1.8)	0.352
Total cholesterol, mmol/l	5.7 (1.6)	5.7 (1.6)	217.9 (5.6)	0.456
Triglycerides, mmol/l	2.1 (0.8)	2.1 (0.8)	2.1 (0.8)	0.570
HDL, mmol/l	1.1 (0.2)	1.1 (0.2)	1.1 (0.2)	0.347
LDL, mmol/l	4.4 (1.3)	4.4 (1.3)	4.4 (1.3)	0.572
Systolic blood pressure, mmHg	137.4 (21.4)	138.3 (22.4)	136.7 (20.6)	0.064
Diastolic blood pressure, mmHg	85.6 (7.2)	86.0 (7.6)	85.4 (6.8)	0.015
Added sugar intake, g/day	60.6 (22.4)	61.4 (23.1)	60.0 (21.9)	0.337
Fruits consumption, g/day	163.2 (49.3)	164.2 (49.4)	162.4 (49.2)	0.558
Vegetable consumption, g/day	282.3 (59.6)	281.0 (60.2)	283.1 (59.4)	0.585

[a] Data are presented as mean (SD) for continuous measures and n (%) for categorical measures; [b] NCDs: non-communicable diseases such as stroke, cardiovascular disease, cancer, diabetes mellitus, and hypertension for father and mother together; NIS, New Israeli Shekel; BMI, Body mass index; HbA$_{1c}$, Haemoglobin A$_{1c}$; HDL, High-density lipoprotein; LDL, Low-density lipoprotein; T2D, type 2 diabetes.

Table S2. Adjusted odds ratio (95 % CI) of the T2D for the sample population participants by average daily added sugar intake (*n* = 1000).

Added sugar intake level g/day	No. of participants *n* (%)	Model 1	Model 2	Model 3	Model 4
<50.0 g/day	216 (21.6%)	1.00	1.00	1.00	1.00
50.0–66.0 g/day	343 (34.3%)	1.26 (0.89–1.78)	1.28 (0.89–1.82)	1.31 (0.92–1.86)	1.28 (0.89–1.81)
67.0–99.0 g/day	361 (36.1%)	0.96 (0.68–1.37)	1.00 (0.69–1.44)	1.02 (0.71–1.47)	1.00 (0.69–1.42)
≥100.0 g/day	80 (8.0%)	1.42 (0.84–2.39)	1.50 (0.88–2.58)	1.54 (0.89–2.64)	1.55 (0.89–2.68)
p for trend		0.556	0.611	0.324	0.338

Model 1, Adjusted for age, sex, population, region, income, education, and smoking; Model 2, Model 1 plus physical activityand body mass index; Model 3, Model 2 plus fruits and vegetable intake; Model 4, Model 3 plus hypertension and lipid profile (total cholesterol, triglycerides, HDL, and LDL).

Table S3. Adjusted OR (95 % CI) of undiagnosed T2D by added sugar intake level after including HbA_{1c} in the definition of outcome (*n* = 548).

Added sugar intake level g/day	No. of participants *n* (%)	Model 1	Model 2	Model 3	Model 4
<50.0 g/day	126 (23.0%)	1.00	1.00	1.00	1.00
50.0–66.0 g/day	181 (33.0%)	2.21 (1.26–3.89)	2.09 (1.18–3.69)	2.07 (1.17–3.68)	2.05 (1.16–3.65)
67.0–99.0 g/day	201 (36.7%)	1.76 (0.99–3.12)	1.56 (0.87–2.79)	1.56 (0.87–2.79)	1.54 (0.86–2.76)
≥100.0 g/day	40 (7.3%)	3.65 (1.59–8.37)	2.91 (1.25–6.81)	2.86 (1.22–6.71)	2.98 (1.27–6.98)
p for trend	<0.001	<0.001	<0.001	<0.001	

Model 1, Adjusted for age, sex, population, region, income, education, and smoking; Model 2, Model 1 plus physical activity and body mass index; Model 3, Model 2 plus fruits and vegetable intake; Model 4, Model 3 plus hypertension and lipid profile (total cholesterol, triglycerides, HDL, and LDL).

Table S4. Adjusted OR (95 % CI) of undiagnosed T2D by added sugar intake level after including HbA_{1c} in the definition of outcome (*n* = 548).

Variable N (%)	<50.0 g/day OR (95% CI) *n* (%)	50.0–66.0 g/day OR (95 % CI) *n* (%)	≥67.0 g/day OR (95 % CI) *n* (%)	*p*-Value $_{trend}$	*p*-Value $_{interaction}$
Sex					0.188
Men 287 (52.4%)	1.00 64 (22.3%)	1.58 (0.70–3.58) 89 (13.0%)	1.91 (0.88–4.14) 134 (46.7%)	0.082	
Women 261 (47.6%)	1.00 62 (23.8%)	2.59 (1.16–5.75) 92 (35.2%)	1.36 (0.61–3.01) 107 (41.0%)	0.942	
Age (Median)					0.372
<59 years 262 (47.8%)	1.00 72 (27.5%)	1.74 (0.73–4.11) 77 (29.4%)	1.61 (0.72–3.63) 113 (43.1%)	0.071	
≥59 years 286 (52.2%)	1.00 54 (18.9%)	1.52 (0.67–3.43) 104 (36.4%)	1.07 (0.47–2.43) 128 (44.7%)	0.686	
BMI					0.001
<26.7 kg/m^2 271 (49.4%)	1.00 64 (23.6%)	2.35 (0.92–6.02) 94 (34.7%)	2.19 (0.86–5.51) 113 (41.7%)	0.460	
≥26.7 kg/m^2 277 (50.6%)	1.00 62 (22.4%)	7.06 (2.77–18.01) 87 (31.5%)	5.02 (2.03–12.42) 128 (46.2%)	0.024	
Population					0.035
Refugees 224 (40.9%)	1.00 46 (20.5%)	5.85 (1.86–18.39) 79 (35.3%)	5.32 (1.70–16.63) 99 (44.2%)	0.028	
Citizens 324 (59.1%)	1.00 80 (24.7%)	5.49 (1.78–16.95) 102 (31.5%)	4.12 (1.36–12.45) 142 (43.8%)	0.849	

Variables adjusted for age, sex, population, region, income, education, smoking, physical activity, body mass index, fruits and vegetable intake, hypertension, and lipid profile (total cholesterol, triglycerides, HDL, and LDL).

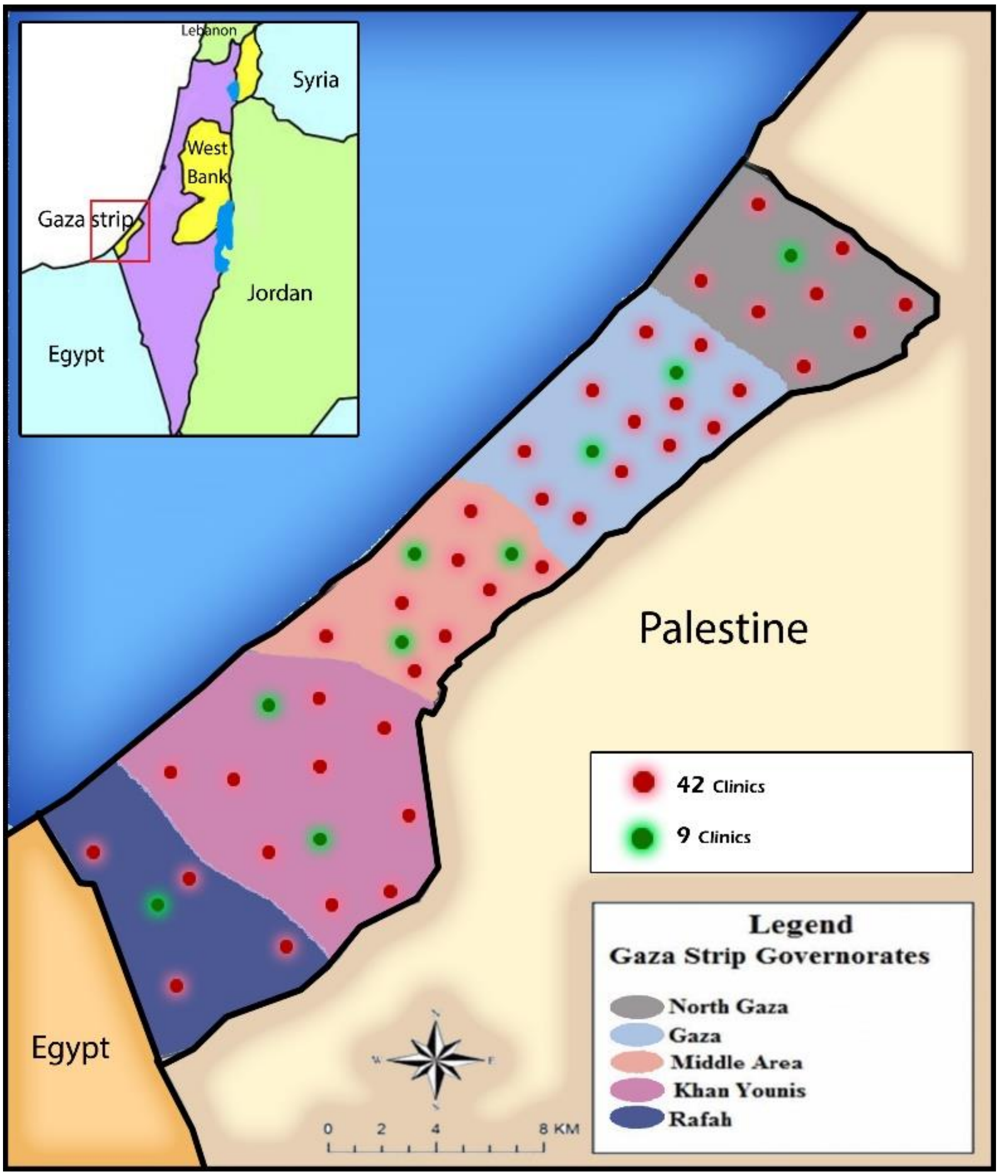

Figure 1. The selected Primary Healthcare Centers (PHCs) across the Gaza Strip governorates (designed by the author).
